# Spectrum of central nervous system infections in a tertiary health care centre in Cameroon

**DOI:** 10.1186/s41983-022-00454-0

**Published:** 2022-02-05

**Authors:** Daniel Gams Massi, Marcel Roger Rodrigue Mintyene Mintyene, Annick Mélanie Magnerou, Seraphine Mojoko Eko, Caroline Kenmegne, Salomon Mbahe, Prince Eliot Sounga Bandzouzi, Hugo Bertrand Mbatchou Ngahane, Njankouo Yacouba Mapoure

**Affiliations:** 1 Faculty of Health Sciences, University of Buea, Buea, Cameroon; 2 Internal Medicine Department, Douala General Hospital, PO Box: 4856, Douala, Cameroon; 3 Faculty of Medicine and Pharmaceutical Sciences, University of Douala, Douala, Cameroon; 4 Internal Medicine Department, Douala Gyneco-Obstetric and Paediatric Hospital, Douala, Cameroon; 5 Department of Neurology, Marien Ngouabi University, Brazzaville, Republic of Congo

**Keywords:** CNS infection, HIV, Cerebral toxoplasmosis, Douala, Cameroon

## Abstract

**Background:**

Central nervous system (CNS) infections are serious and debilitating diseases with significant mortality, and high prevalence in the context of human immunodeficiency virus (HIV) pandemic in Africa. However, their diagnosis remains challenging due to outdated technical platform. We aimed to determine the frequency of CNS infection and to describe the epidemiological, clinical and outcome of this at the Douala General Hospital (DGH), Cameroon. To carry out this study, we collected the medical records of patients hospitalized for CNS infections in the internal medicine department of DGH from January 2015 to December 2019.

**Results:**

Among 8430 files reviewed, 336 cases of CNS infection were identified giving a frequency of CNS infection of 3.99% among which 204 files were included in the study (54.4% were male). HIV infection was found in 147 patients (72.1%) with 38.1% (*n* = 56) of them on regular follow-up. The most common clinical signs were fever (84.8%), headache (68.6%), meningeal syndrome (38.7%), and seizures (36.3%). Cerebral toxoplasmosis (24.5%), cryptococcal meningitis (21.1%), and acute bacterial meningitis (8.3%) were leading aetiologies. Of the 143 CSF samples, 70.6% (*n* = 101) were sterile. The in-hospital mortality rate was 23.5% with CNS infection of unknown cause (22.1%) be independently associated to this [OR = 2.24; 95% CI 1.04–4.80, *p* = 0.039].

**Conclusion:**

Clinical presentations of CNS infections are same with classical data. HIV-related opportunistic infections are the main aetiologies. About one over four patients with CNS died. Two thirds of CSF are sterile using basic laboratory assessment giving a need to identify simple tests to increase sensibility and specificity of diagnostic tools in our setting.

## Background

Central nervous system (CNS) infections are potentially life-threatening conditions that constitute a diagnostic and therapeutic emergency [1–5]. It is a group of neurological conditions with varying symptoms depending on the type of infection, the pathogen involved and the site in which they occur [4, 6]. These infections are caused by microorganisms (viruses, bacteria, fungi, protozoa); macro-organisms and microparticles (prions) [3, 6–8]. The causative organisms vary depending on their location in the CNS, geographic exposure, vaccination status, age, surgical procedures and immune status [2, 8]. Knowledge of the most common aetiologies is important for the appropriate choice of antimicrobial therapy, which must be instituted promptly to reduce morbidity and mortality and prevent long-term neurological sequelae [2, 3]. CNS infections are relatively rare in developed countries but remain a public health problem in developing countries [1, 9, 10].

The global incidence of CNS infections between 1990 and 2016 is estimated at 389/100,000 [12]. In Africa, pooled data on CNS infections are scarce. Meningitis epidemics have, however, been declared in the countries of the Sub-Saharan meningitis belt [13–16]. In Nigeria, 393,614 cases of meningitis were reported between 1991 and 2011 by the World Health Organization (WHO) [17]. Immunosuppression is frequently associated to CNS infections. Thus, patients infected with the Human Immunodeficiency Virus (HIV) will be more exposed to these infections [18–20]. Between 2006 and 2016, 102 cases of cryptococcal meningitis were reported in Togo [20]. In Cameroon, between 2004 and 2009, cerebral toxoplasmosis (32%) and cryptococcal meningitis (25%) were reported as commonest CNS infections in HIV immunocompromised patients [19]. However, *Streptococcus pneumonia* (46%) and *Neisseria meningitis* (30%) were the main aetiologies among 135 cases of meningitis reported in Cameroon [21].

Morbidity and mortality associated with CNS infections is higher compared to infections of other systems [5]. Despite remarkable progress in their prevention and management, CNS infections remain a major cause of death, particularly in developing countries and in immunocompromised patients [7]. Without treatment, the mortality from bacterial meningitis is close to 100%, while with adequate treatment the mortality is reduced to 4.2% [22]. The overall mortality from viral encephalitis is estimated between 3.8% and 7.4% with an incidence of 2.2/100,000 in adults and 6.3/100,000 at any age [23]. This study aims to provide data in a resource limited area for decision-making and improvement of the management of this public health concern.

## Methods

A retrospective cohort study was conducted in the internal medicine department of the Douala General Hospital (DGH). Medical files were reviewed for patients aged 16 years and above, hospitalized between January 1, 2015 and December 31, 2019 for CNS infection diagnosed via neuroimaging (brain CT scan and MRI) with and without cerebrospinal fluid (CSF) analysis. All patients performed at least brain CT scan/MRI and/or CSF analysis. Each file stored in the archives was recorded in a register, as well as in a computer which contained the hospital reports of each patient. Incomplete files, especially without neuroimaging and/or CSF analysis results were excluded. Ethical approval was obtained from institutional ethics committee of the University of Douala (No. 2162CEI-UDo/01/2020/T).

The following data were collected from selected medical files: (1) sociodemographic data: age, gender, profession, marital status; (2) comorbidities: HIV, diabetes, hypertension, chronic kidney disease (CKD); (3) clinical data: neurological and extra-neurological signs; (4) paraclinical workups: neuroimaging (brain CT scan or MRI), CSF analysis when done, and other biological testing, such as CBC, CRP, blood culture; and (5) outcome: mean length of hospitalization and mortality.

The CSF analysis carried out by the laboratory of the DGH are classically: biochemistry (protein and glucose level), cell count (leucocytes and red blood cells), direct detection (Gram stain, India ink, and Ziehl–Neelsen stain), culture on blood or chocolate agar (bacteria), Lowenstein–Jensen medium (*Mycobacterium tuberculosis*), Sabouraud dextrose agar (fungi), soluble bacterial antigens, cryptococcal antigens, and Veneral disease research laboratory (VDRL)/*Treponemal Pallidum* Hemagglutination assay (TPHA). When requested, a real-time polymerase chain reaction (PCR) for *Mycobacterium tuberculosis* also called Xpert MTB/RIF Molecular Beacon Assay was performed at the laboratory of the Regional Delegation of Public Health of the Littoral. Hypoglycorachia was defined as a CSF glucose level less than half of the glycaemia; hyperproteinorachia was defined as a CSF protein level in the CSF greater than 0.45 g/L; and pleocytosis was considered as a white cell count > 5 cells/mm^3^.

### Statistical analysis

The data collected was recorded in our database and was analysed using SPSS software version 23.0. Categorical variables were expressed as frequencies with percentages. Continuous variables were presented as mean ± standard deviation (SD). χ^2^ and Student tests were used to compare categorical and continuous variables, respectively. Multivariate analysis using logistic regression was performed to determine factors associated to death. Results were considered significant for a *p*-value < 0.05.

## Results

**Fig. 1:**
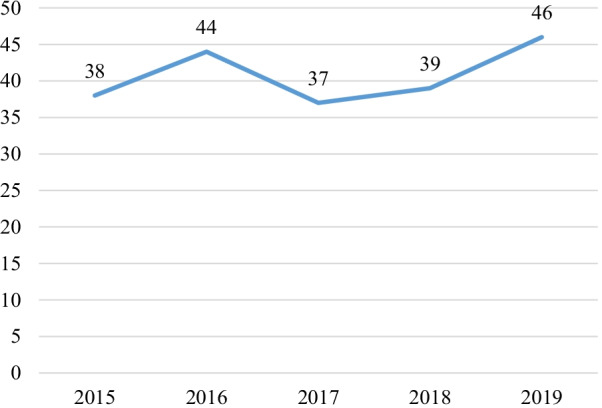
Trends of CNS infection from 2015 to 2019

Out of the 8430 files reviewed, 336 cases of CNS infection were identified, of which 204 met the inclusion criteria. From 2015 to 2019, the hospital-based frequency of CNS infections ranged from 2.2% to 2.6% (Fig. 1).

The mean age of the patients was 42.6 ± 13.79 years (16–84 years). The most common age groups were 30–40 years (29.9%) and 40–50 years (28.4%). Male patients accounted for 54.4% of cases. HIV infection was found in 147 cases (72.1%) with 61.9% of them not on regular follow-up with highly active antiretroviral therapy (HAART) (Table 1).

**Table 1:** Risk factors and comorbidities

Variables	Followed-up	Not followed-up	Total (%)
HIV	91	56	72.1
Alcohol	58	–	28.4
Other surgery	15	–	7.4
Smoking	12	–	5.9
Hypertension	9	21	14.7
Diabetes	6	4	4.9
CKD	3	8	5.4
Brain surgery	1	–	0.5

*CKD* chronic kidney disease, *HIV* human immunodeficiency virus

The most common neurological signs were headache (68.6%), impaired consciousness (44.1%), signs of meningeal irritation (38.7%), and epileptic seizures (36.3%), while altered general state (93.1%), fever (84.8%) and vomiting (35.8%) were main extra-neurological signs (Table 2).

**Table 2:** Clinical manifestations in patients with CNS infections

Clinical signs	*n*	%
Neurological signs		
Headaches	140	68.6
Impaired consciousness	90	44.1
Meningeal signs	79	38.7
Seizures	74	36.3
Focal neurological deficits	59	28.9
Extra-neurological signs		
Altered general state	190	93.1
Fever	173	84.8
Vomiting	73	35.8
Respiratory distress	52	25.5
Gastro-intestinal tract signs	42	20.6
Dehydration	28	13.7
Clinical anaemia	17	8.3
Skin rash	7	3.4

**Fig. 2:**
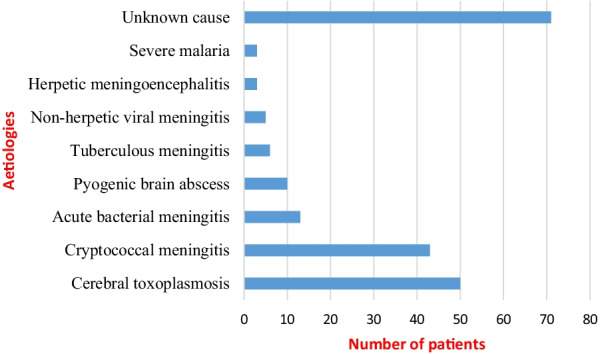
Aetiologies of CNS infections

Neuroimaging was performed in 177 patients (brain CT scan = 131; brain MRI = 46). Results were abnormal in 117 cases with brain mass representing the commonest lesion reported. These abnormalities were found in 65.6% and 67.4% of brain CT scan and brain MRI, respectively. Lumbar puncture with CSF analysis was performed in 143 patients. Results showed hypoglycorachia (*n* = 67; 46.6%), hyperproteinorachia (*n* = 103; 77.4%), and pleiocytosis (*n* = 42; 29.4%). CSF microbiological analysis was positive for *Cryptococcus neoformans* (*n* = 38), *Neisseria meningitidis* (*n* = 1), *Streptococcus pneumoniae* (*n* = 1) and *Pseudomonas aeruginosa* (*n* = 1). No acid-fast bacilli were detected through the Ziehl–Neelsen stain. PCR was positive for *Mycobacterium tuberculosis* in three cases. Among the patients with HIV, the mean CD4 count (in 94 patients) was less than 350 cells/mm^3^ in 92.3% of cases. The most frequent aetiologies were cerebral toxoplasmosis (24.5%) and cryptococcal meningitis (21.1%), while unknown aetiology was founded in 34.8% of cases (Fig. 2).

The mean length of hospitalization was 9.5 ± 7.1 days with an in-hospital mortality was 23.5%. In the univariate analysis, factors associated with in-hospital mortality were: age groups of 30–40 (*p* = 0.012), 40–50 (*p* = 0.011) and CNS of unknown aetiology (*p* = 0.006). After multivariate analysis, only CNS infection of unknown aetiology was independently associated to the in-hospital mortality (OR = 2.24, 95% CI 1.04–4.8), *p* = 0.039) (Table 3).

**Table 3:** Predictive factors of mortality

Variables	Crude OR (95% CI)	*p* value	Adjusted OR (95% CI)	*p* value
Age [20–30]	Ref.	–	–	–
Age [30–40]	0.16 (0.04–0.64)	0.012	0.75 (0.24–2.52)	0.632
Age [40–50]	0.15 (0.03–0.64)	0.011	0.69 (0.22–2.33)	0.535
Age [50–60]	0.31 (0.06–1.14)	0.135	1.30 (0.36–4.89)	0.686
Age [60–70]	0.25 (0.05–1.31)	0.102	0.80 (0.18–3.40)	0.759
Age ≥ 70	–	–	3.38 (0.63–20.33)	0.164
Female	Ref.	–	–	–
Male	1.10 (0.57–2.11)	0.770	–	–
HIV	0.55 (0.27–1.10)	0.094	1.04 (0.42–2.71)	0.927
Diabetes	0.80 (0.16–3.92)	0.788	–	–
Hypertension	1.48 (0.62–3.5)	0.368	–	–
CKD	2.90 (0.84–9.98)	0.090	2.05 (0.50–7.98)	0.300
CD4 count ≤ 200/mm^3^	1.6 (0.33–7.56)	0.553	–	–
Cerebral toxoplasmosis	0.21 (0.07–0.64)	0.006	–	–
Cryptococcal meningitis	1.56 (0.73–3.30)	0.246	–	–
Bacterial meningitis	1.23 (0.31–4.84)	0.764	–	–
Unknown aetiology	2.52 (1.30–4.88)	0.006	2.24 (1.04–4.80)	0.039

*CKD* chronic kidney disease, *HIV* human immunodeficiency virus

## Discussion

This study reviewed cases of CNS infections in a referral hospital in Cameroon and aimed to determine his frequency and to describe their characteristics. There was a slight increase in the number of cases reported from 2015 to 2019. HIV was the main comorbidity associated to CNS infections. The main aetiologies were HIV related opportunistic CNS infections. The in-hospital mortality was significant.

In Sub-Saharan Africa (SSA), CNS infections are frequent causes of admission in neurology [24]. The overall intra-hospital prevalence of CNS infections in this study was underestimated. A recent study done in Cameroon including patients from outpatient department and hospitalization in two referral hospitals reported a prevalence of 11.3% in 2018 in Cameroon [11]. The flow of patients hospitalized for CNS infection does not reflect the reality.

Young adults represented the main age group. Several studies in Africa reported similar data [19, 25–27]. In Europe, Erdem et al. found a mean higher than our findings [28]. This gap could be explained by the difference in demographical characteristics of African and European populations. In addition, the most frequent age groups in this study are similar to the HIV most affected age groups in the Cameroonian population (Cameroon population-based HIV impact assessment, 2017). The male predominance found in this study was consistent with other studies [26, 29–31]. Genetic and hormonal difference may contribute to this gender difference. Oestrogens was described to play a protective role against infections by reducing the inflammatory response [32]. Close to three quarters of patients were PLWH. Similar results were reported in Ivory Coast and Mali [27, 33].

Clinical manifestations are often similar to those of classical data [4]. In this study, the neurological signs were mainly: headache, impaired consciousness, meningeal syndrome, and seizures. Diarra et al. found meningeal syndrome in 34.5% of cases [27]. Meningeal syndrome is a clinical expression of the inflammatory reaction produced when the meninges are invaded by a pathogen. Thus, a frank meningeal syndrome would reflect a significant inflammatory response of the CNS. The population in this study is characterized by a high frequency of HIV infection low immunity (CD4 cells less than 350/mm^3^). This could explain the lower inflammatory response, hence the low frequency of patients with meningeal syndrome [34].

The leading aetiologies were cerebral toxoplasmosis (24.5%), cryptococcal meningitis (21.1%), and acute bacterial meningitis (5.9%). These findings are consistent with a study done by Luma et al. in 2013, which found cerebral toxoplasmosis (32.3%) and cryptococcal meningitis (25%) as the commonest aetiologies [19]. Given the endemic context of HIV and the high frequency of people living with HIV (PLWH) in our context, opportunistic infections such as cerebral toxoplasmosis and cryptococcal meningitis remain very frequent [35]. The occurrence of opportunistic infections is favoured by the low rate of patients with HIV under regular HAART in this population. In more than one-third of cases, the aetiology remains undetermined compared to 8.8% of cases reported in Europe [28]. This diagnostic gap reflects the difficulty encountered in resource-limited settings [3]. These difficulties are accentuated by the fact that more than a quarter of these patients are referred from primary health centres, where patients receive empirical antibiotics without any microbiological evidence. More efficient diagnostic tools may contribute to improve the identification of pathogens.

In 2013, Luma et al. reported a mortality of 49% in PLWH which is more than two time higher than our finding [19]. Several studies have reported mortality rates greater than 10% [21, 26, 27, 36]. Cerebral toxoplasmosis represented the lowest case fatality rate in this series. In multivariate analysis, there was a statistically significant association between the occurrence of death and the undetermined aetiologies (adjusted OR = 2.24; *p* = 0.039). This could be explained by the fact that without knowing of the causes, specific treatment cannot be instituted, thereby increasing the risk of death [1].

This study places the foundations for epidemiological data on CNS infections in Cameroon. However, the retrospective design of this study leads to data collection challenges with up to 132 incomplete medical files excluded. This could have impacted the reliability of results. In addition, this study was conducted in one hospital, and therefore, data cannot be generalized in the entire community.

## Conclusions

Clinical presentations of CNS infections are same with classical data. HIV-related opportunistic infections are the main aetiologies. Mortality remains high in our context. Two over third CSF are sterile using basic laboratory assessment giving a need to implement simple tests to increase sensibility and specificity of diagnostic tools in our setting.

## Data Availability

Data can be made available upon reasonable request.

## Abbreviations

CNS: Central nervous system
HIV: Human immune-deficiency virus
DGH: Douala general hospital
CSF: Cerebrospinal fluid
WHO: World Health Organization
CT: Computed tomography
MRI: Magnetic resonance imaging
CKD: Chronic kidney disease
CBC: Complete blood count
CRP: C-reactive protein
VDRL: Veneral disease research laboratory
TPHA: Treponemal Pallidum Hemagglutination assay
PCR: Polymerase chain reaction
MTB/RIF: Mycobacterium tuberculosis DNA and resistance to rifampicin
SD: Standard deviation
HAART: Highly active antiretroviral therapy
PLWH: People living with human immune-deficiency virus
